# Influence of fat infiltration, tear size, and post-operative tendon integrity on muscle contractility of repaired supraspinatus muscle

**DOI:** 10.1007/s00590-021-03020-1

**Published:** 2021-06-19

**Authors:** Takuma Yuri, Nariyuki Mura, Kyosuke Hoshikawa, Hugo Giambini, Hiromi Fujii, Yoshiro Kiyoshige

**Affiliations:** 1 Graduate School of Health Sciences, Yamagata Prefectural University of Health Sciences, 260 Kamiyanagi, Yamagata, 990-2212 Japan; 2 Department of Biomedical Engineering, The University of Texas At San Antonio, San Antonio, TX USA; 3 Department of Orthopaedic Surgery, Yoshioka Hospital, Tendo, Japan; 4 Department of Physical Therapy, Yamagata Prefectural University of Health Sciences, Yamagata, Japan

**Keywords:** Contractility, Rotator cuff tear, Goutallier stage, Cofield classification, Sugaya classification, Real-time tissue elastography

## Abstract

**Background:**

The purpose of this study was to evaluate the effect of fat infiltration, tear size, and post-operative tendon integrity, on post-operative contractility.

**Methods:**

Thirty-five patients who underwent rotator cuff repair were included. The fat infiltration, tear size, and post-operative tendon integrity were evaluated by Goutallier stage, Cofield classification, and Sugaya classification, respectively. The muscle elasticity at rest and at contraction was assessed by real-time tissue elastography pre- and one-year post-operatively. We defined the difference in elasticity between at rest and at contraction as the activity value which reflects muscle contractility.

**Results:**

The activity value in patients with Sugaya *Type I* tended to increase regardless of Cofield classification, whereas those with Sugaya *Type III* and *IV* tended to decrease. While the activity value in the patients classified as stage 1 and *Type I* tended to increase, patients classified as stage 2 showed decreased or constant in contractility even in those subjects classified as *Type I*. Stepwise multiple regression analysis showed both pre- (*p* = 0.004, *r* = -0.47) and post-operative activity values (*p* = 0.022, *r* = -0.39) to be significantly correlated only with the Goutallier stage.

**Conclusion:**

Multiple regression analysis indicated only the Goutallier stage was a significant independent factor for contractility of the supraspinatus muscle. Supraspinatus muscle contractility in patients classified as *Types III* and *IV* based on the Sugaya classification tended to decrease post-operatively, while patients whose contractility increased post-operatively were characterized by having a *Type I* tendon integrity.

## Introduction

Rotator cuff tears are a common problem that results in functional limitations [1]. While fat infiltration, tear size, and post-operative tendon integrity were considered to have a significant effect on post-operative functional outcomes in the patients with rotator cuff tear, these three prognostic factors may affect each other, still remaining a controversial topic as it relates to etiology and functional outcomes [2, 3].

The supraspinatus is the most common rotator cuff tendon that is involved in a rotator cuff tear [4–6]. Yuri et al. demonstrated that the pre-operative contractility of the torn supraspinatus muscle can be measured with real-time tissue elastography (RTE), non-invasively and quantitatively [7]. While functional recovery of the supraspinatus muscle itself is not responsible for entire shoulder functional outcome, it will provide us with important information in the setting of rotator cuff tear and repair. However, to our knowledge, there are no study investigating the recovery of contractility itself. Quantitative relationship between fat infiltration, tear size, tendon integrity, and post-operative contractility may need to be considered when clinicians choose interventions for patients with rotator cuff tear. The purpose of this study was to elucidate pre- and post-operative contractility of the supraspinatus muscle, and evaluate the effect of prognostic factors, such as fat infiltration, tear size, and tendon integrity, on post-operative contractility. We hypothesized that pre-operative fat infiltration could be a significant indicator for post-operative contractility of supraspinatus muscle.

## Materials and Methods

### Participants

Thirty-five patients (mean age 67 years old; 22 males and 13 females) who underwent double row (suture-bridge technique) and following conventional rehabilitation protocol, as well as both pre- and one-year post-operative measurements, were included in this study after approval from our Ethics Committee. Written informed consent to participate in this study was obtained from all patients. The time from injury onset to tendon injury repair ranged from 1 to 36 months. Tear size was intra-operatively classified based on the Cofield’s classification [8]: *small* (one centimeter or less); *medium* (one to three centimeters); *large* (three to five centimeters); and *massive* (greater than five centimeters). Fat infiltration of the supraspinatus muscle was evaluated on the sagittal Y-view, where the most lateral image on which the scapular spine was in contact with the rest of the scapula, using magnetic resonance imaging (MRI) and the Goutallier stage[9, 10]: *stage 1* (the muscle contains some fatty streaks); *stage 2* (fat infiltration is important but there is still more muscle than fat); *stage 3* (there is as much fat as muscle); and *stage 4* (more fat than muscle is present). Repaired supraspinatus tendon integrity was assessed on the coronal plane of the MRI based on the Sugaya classification [11]: *Type I* (sufficient thickness with homogeneous low intensity); *Type II* (sufficient thickness with partial high intensity); *Type III* (insufficient thickness without discontinuity); *Type IV* (presence of a minor discontinuity); and *Type V* (presence of a major discontinuity).

### Contractility Measurements

**Fig. 1:**
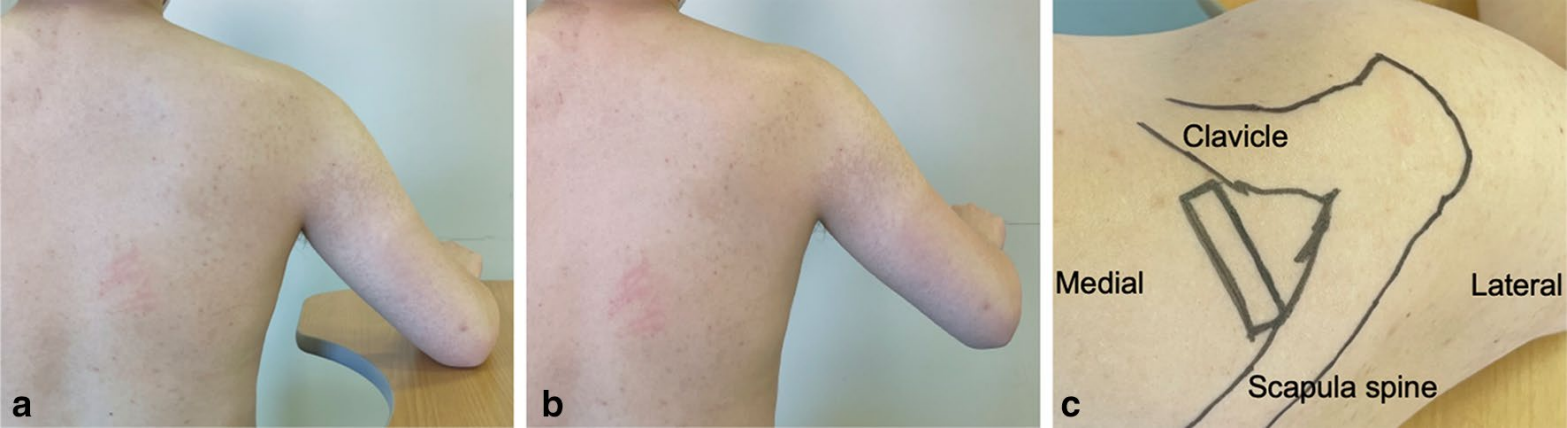
Contractility measurement procedures **a** Patient position during at rest measurement. The patient sat on a chair with the affected arm resting on a table with 60 degrees abduction in the scapular plane. **b** Patient position during contraction measurement. The patient held his affected arm against gravity. **c** Superior view of the right shoulder. Cross-sectional ultrasound B-mode images were oriented by placing the probe 2-cm medial to the acromion process

The RTE measurements were performed on the day before surgery and one-year after surgery [7]. A diagnostic ultrasound system (Noblus; Hitachi-Aloka Medical Japan, Tokyo, Japan) with a linear array probe (L-64; Hitachi-Aloka Medical Japan) combined with an acoustic coupler (EZU-TECPL1; Hitachi-Aloka Medical Japan: 22.6 ± 2.2 kPa) was used to obtain the measurements. Briefly, the patient was first instructed to sit on a chair with the affected arm resting on a table, then the shoulder was abducted to 60 degrees in a neutral rotation with respect to scapular plane (Fig. 1a and b). Cross-sectional ultrasound B-mode images were oriented by placing the probe 2-cm medial to the acromion process (Fig. 1c), and elastography images were obtained by performing cyclic manual compression with the probe. The patient was assessed by RTE while at rest and at contraction consisting on the patient holding the affected arm against gravity for ten seconds.

After continuous scanning, three random images at rest and at contraction were selected to calculate mean ± SD. The elasticity from the whole cross-sectional area was calculated as the strain ratio (SR) which describes the relative value in reference to hardness of the acoustic coupler. Two SR values were defined: SR value at rest and SR value at contraction. Because the former is an indicator of the muscle elasticity at rest, while the latter represents the sum of muscle elasticity at rest and the elasticity produced by contraction, we defined the difference between SR value at rest and SR value at contraction as the activity value, with this reflecting muscle contractility [7, 12, 13]. When the activity values were low, the muscle acted as a dynamic tenodesis, while those were high, the muscle acted as power production through substantial voluntary contractions. Total measurement and analysis times were less than 15 min.

### Statistical Analysis

The SPSS statistical software (version 24.0; SPSS, Chicago, IL, USA) was used for data analyses. Intra-class correlation coefficient (ICC_1,3_) was calculated using elasticity measurements to evaluate variability in measurements. Data evaluation using the Shapiro–Wilk test indicated a non-normal distribution; thus, non-parametric tests were subsequently performed where appropriate. Differences between post-operative contractility in patients with *Type I* Sugaya classification and those with *Type III* were tested by the Mann–Whitney U test. The effect of the Cofield classification and the Goutallier stage on post-operative activity value was tested using the Kruskal–Wallis test, followed by the Bonferroni post hoc test. The Wilcoxon test was used to compare pre- and post-operative activity values. Finally, stepwise multiple regression analysis was used to investigate whether age, sex, time from injury onset, Sugaya and Cofield classifications, and Goutallier stage as independent variables could explain the activity value. A *P-*value less than 0.05 was considered as statistically significant.

Using G power 3.1 software, we conducted post hoc power analysis with an *α* of 0.05. The power for a stepwise multiple regression analysis was 0.63. The power of subgroup analysis was underpowered (< 0.20), except for the comparison of pre- and post-operative activity value in the patients with stage 1 (0.66).

## Results

The distribution of tear size in our patient cohort was as follows: 3 small, 20 medium, 8 large, and 4 massive. Goutallier stage classification resulted in 3 stage 0, 25 stage 1, 6 stage 2, and 1 stage 3. Degree of fat infiltration showed no changes post-operatively compared to pre-operative evaluation. Sugaya classification resulted in 26 *Type I*, 1 *Type II*, 7 *Type III*, and 1 *Type IV*.

**Fig. 2:**
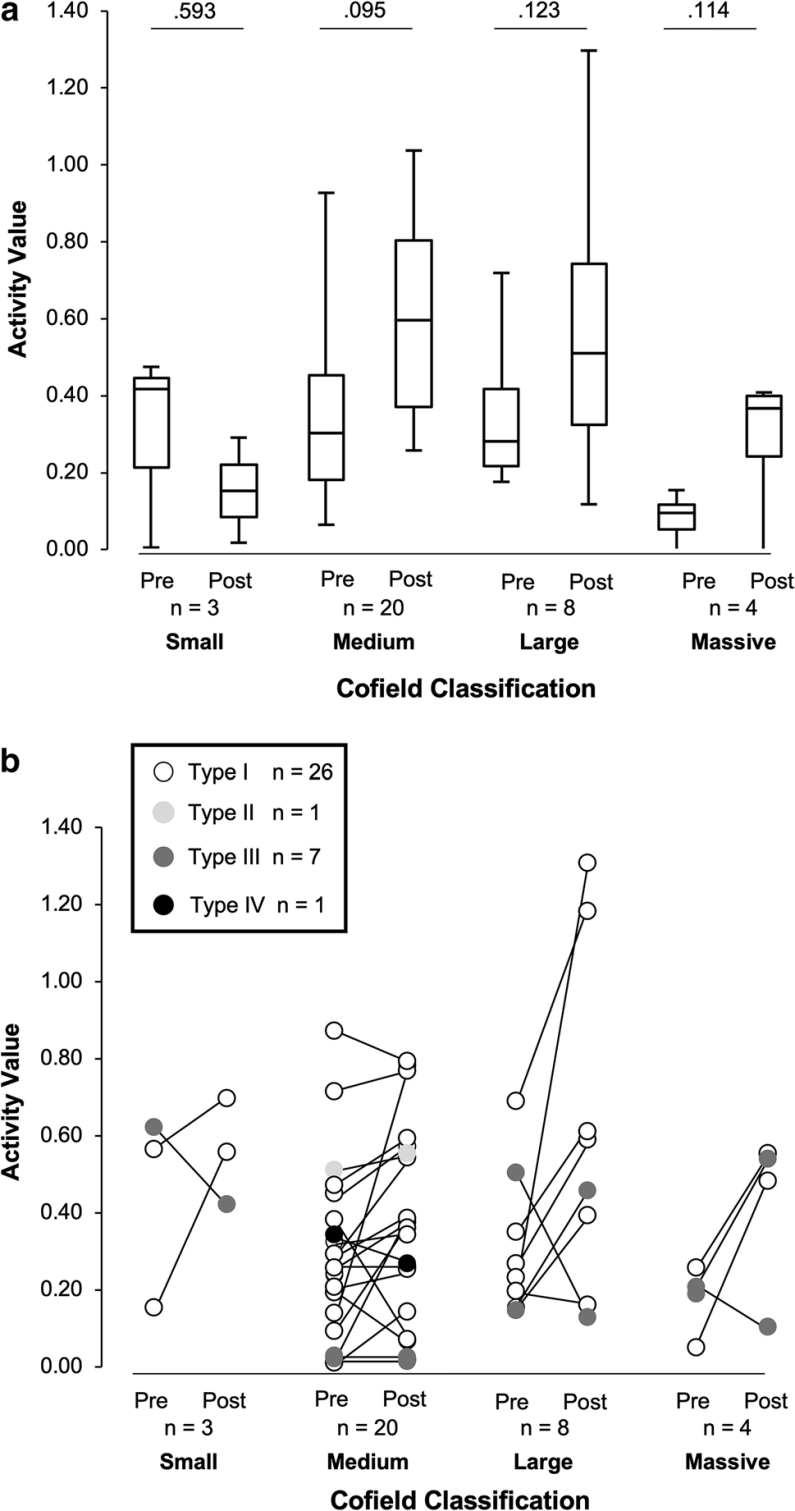
**a** Pre- and post-operative activity values in relation to the Cofield classification. **b** Colour plots of repaired tendon integrity according to Sugaya classification and muscle activity versus the Cofield classification

**Fig. 3:**
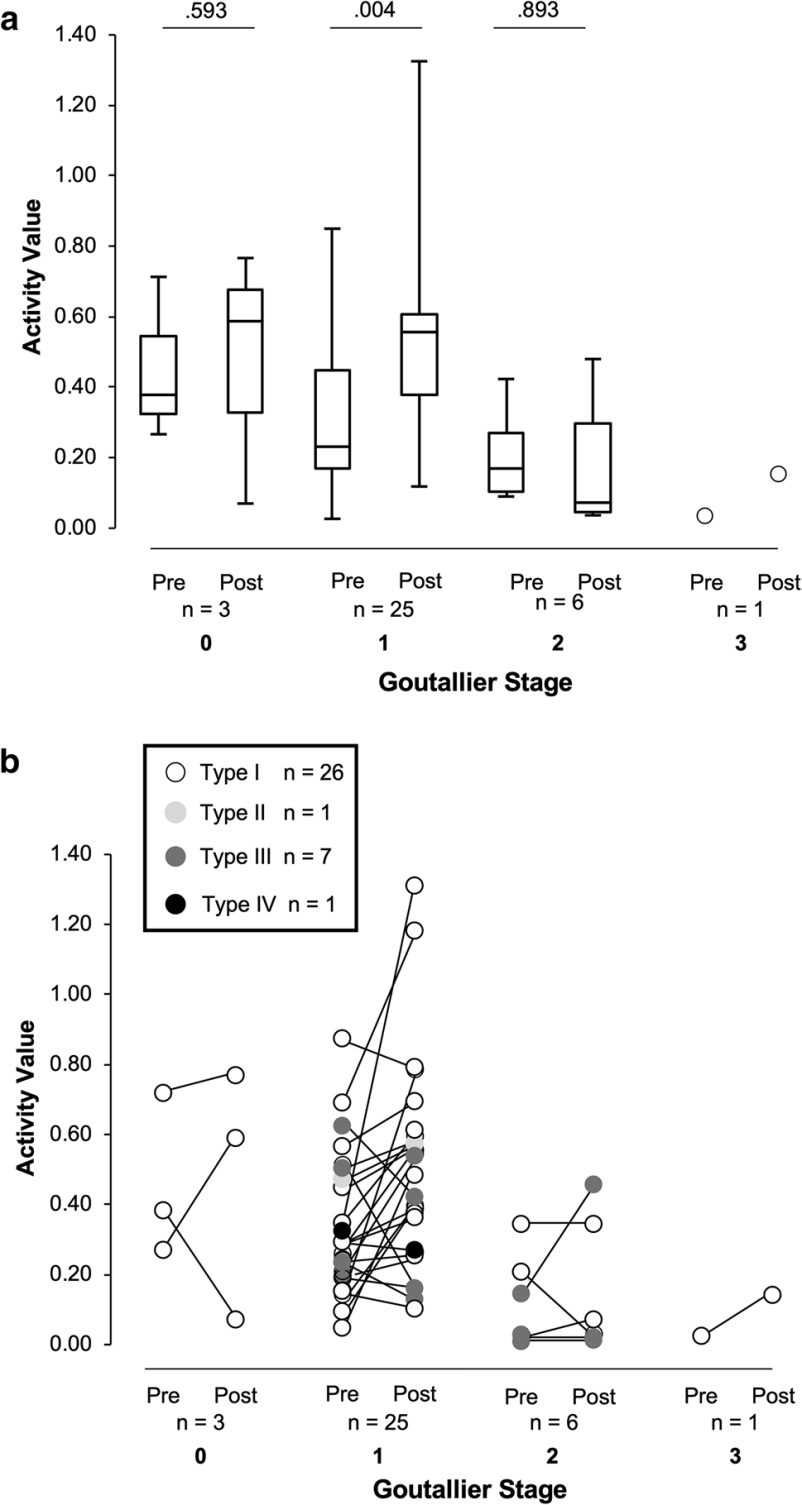
**a** Pre- and post-operative activity values in relation to the Goutallier stage. **b** Colour plots of repaired tendon integrity according to Sugaya classification and muscle activity versus the Goutallier stage

The ICC_1,3_ outcomes for elasticity measurements showed excellent reliability (0.92–0.98). Mean ± SD pre-operative SR values at rest and at contraction, and activity values were 0.34 ± 0.22, 0.04 ± 0.03, and 0.29 ± 0.21, respectively. Post-operative outcomes were 0.46 ± 0.31, 0.02 ± 0.01, and 0.44 ± 0.30, respectively. Table 1 shows pre- and post-operative activity values in relation to Sugaya classification. Overall post-operative activity value was significantly higher than that measured pre-operatively (*p* = 0.004). Post-operative activity values in patients classified as *Type I* were significantly higher than those obtained pre-operatively (*p* = 0.001), while there was no significant difference between pre- and post-operative activity values in patients classified as *Type III* (*p* = 0.612). Post-operative activity values in patients with *Type I* were significantly higher than those in *Type III* (*p* = 0.034; Table 1). The activity values increased in twenty-two (22) out of twenty-six (26) patients (84.6%) with *Type I* tendon integrity, one (1) out of one (1) patient (100.0%) with *Type II*, and two (2) out of seven (7) patients (28.6%) with *Type III* (Figs. 2b, 3b).

**Table 1:** Activity values for the supraspinatus muscle in relation to Sugaya classification

Activity value	Overall patients	Type I	Type II	Type III	Type IV	*p*
	(*n* = 35)	(*n* = 26)	(*n* = 1)	(*n* = 7)	(*n* = 1)	
Pre-operative	0.29 ± 0.21	0.30 ± 0.21	0.47	0.24 ± 0.21	0.32	–
Post-operative	0.44 ± 0.30*	0.49 ± 0.31*	0.55	0.24 ± 0.21	0.26	0.034

*p* refers to differences obtained based on the Mann–Whitney U test between *Type I* and *Type III*. Overall activity values were significantly higher post-operatively (*p* = 0.004). Post-operative activity values in the patients with *Type I* were significantly higher than pre-operative outcomes (*p* = 0.001), while there was no significant difference between pre- and post-operative activity values in patients with *Type III* (*p* = 0.612). * indicate the significant difference between pre- and post-operative outcomes.

Figure 2 shows the pre- and post-operative activity values in relation to the Cofield classification. There were no significant differences between pre- and post-operative activity values in patients with small, medium, large, and massive tears (*p* = 0.593, *p* = 0.095,* p* = 0.123, and *p* = 0.114, respectively; Fig. 2a). Similarly, the Kruskal–Wallis test showed there were no significant differences in post-operative activity value among tear sizes (*p* = 0.300). Figure 2b shows the distribution of the individual patients based on their activity values and the Cofield classification. Patients classified as *Types I* and *III* were distributed among all tear sizes. The activity value in patients with *Type I* increased regardless of tear size, except for two medium and one large tear patients. The activity value in the patients with *Type III* decreased regardless of Cofield classification, except for four patients in medium, large, and massive tears who showed an increase in activity value.

Figure 3 shows pre- and post-operative activity values in relation to Goutallier stage. Post-operative activity values in patients with stage 1 were significantly higher than that observed pre-operatively (*p* = 0.004), while there were no significant differences between pre- and post-operative activity values in patients with stage 0 and 2 (*p* = 0.593 and *p* = 0.893; Fig. 3a). The Kruskal–Wallis test showed there were significant differences in post-operative activity value among the Goutallier stages (*p* = 0.013). Post hoc analysis showed activity value in patients with stage 2 to be significantly lower than those with stage 1 (*p* = 0.011). There were no significant differences in activity value between patients classified as stage 0 and 1 (*p* > 0.999), as well as between stage 0 and 2 (*p* = 0.157). Figure 3b shows four out of twenty-five patients with a stage 1 and three out of six patients with a stage 2 classified as *Type III*. Patients classified as stage 2 showed decreased or constant activity value even in those subjects with good tendon integrity and classified as *Type I,* while the activity value in the patients classified as stage 1 and *Type I* tended to increase.

Finally, stepwise multiple regression analysis showed both pre- and post-operative activity values to be significantly correlated with the Goutallier stage (*p* = 0.004, *r* = − 0.47 and *p* = 0.022, *r* = − 0.39; Table 2).

**Table 2:** Stepwise multiple regression analysis for pre- and post-operative activity values

	Pre-operative activity value		Post-operative activity value	
	*β*	*p*	*β*	*p*
Age	− 0.21	0.240	− 0.12	0.945
Sex	0.04	0.809	0.24	0.165
Onset	0.09	0.598	0.04	0.805
Sugaya’s classification	-	-	− 0.29	0.094
Cofield’s classification	− 0.08	0.667	0.08	0.660
Goutallier’s stage	− 0.47	0.004	− 0.39	0.022
*R*^*2*^	0.22		0.15	
*p*	0.004		0.022	

## Discussion

We measured pre- and post-operative supraspinatus muscle activity value, as a surrogate for contractility, in patients with rotator cuff tears using RTE. The supraspinatus muscle contractility in patients classified as *Types III* and *IV* based on the Sugaya classification tended to decrease post-operatively, regardless of tear size and Goutallier stage; while patients whose contractility increased post-operatively were characterized by having a *Type I* and *II* tendon integrity. Significant differences in post-operative contractility were not observed in the Cofield classification (among small, medium, large, and massive groups; *p* = 0.300). On the other hand, significant post-surgical differences were observed when classifying the shoulders based on the Goutallier stage (among stage 0, 1, and 2; *p* = 0.013) and Sugaya classification (between *Type I* and *III*; *p* = 0.034). As we hypothesized, only the Goutallier stage was a significant independent factor for muscle contractility. To our knowledge, this is the first study to measure post-operative contractility of the repaired supraspinatus muscle in patients with rotator cuff tears.

Goutallier et al. showed that rotator cuff tears lead to muscle degeneration over time and fat infiltration is correlated with severe functional impairment [9, 14]. The authors described stage 2 as a critical point. Other investigators reported a negative relationship between fat infiltration and pre- or intra-operative contractility of the torn supraspinatus muscle [7, 15]. In our study, muscle contractility from shoulders classified as stage 2 were significantly lower than those in stage 1, and a significant increase in contractility between pre- and post-operative measurements was found in stage 1 shoulders but not in stage 2. These outcomes indicate that contractility of shoulders with a Goutallier stage 2 may be highly affected, and the tendon muscle unit classified as stage 2 and 3 may act in dynamic tenodesis manner, if tendon integrity exists.

The Sugaya classification has been widely used to evaluate post-operative repaired tendon integrity [11]. The tendons classified as *Types I* and *II* relate a successful repair, while *Type IV* and *V* define failure of the repair and healing process. However, it is still controversial how *Type III* tendons should be interpreted. Our results show post-operative muscle contractility in *Type I* shoulders to be significantly higher than that of *Type III* shoulders (*p* = 0.034). A significant higher muscle contractility was found in *Type I* (*p* = 0.001) post-operatively, but not in *Type III* (*p* = 0.612). Pre-to-post-operative muscle activity changes in *Type III* shoulders were varied: two increased, three decreased, and two showed no changes. These results suggest that *Type III* shoulders should be considered as an independent category along with the Sugaya classification[11], and that the varied contractility observed in *Type III* could indicate that the post-operative tension, i.e., the attenuation of the repaired tendon, differs between the patients with *Type III*. Adequate tension on the repaired tendon, that present in *Type I* and *II*, could be necessary to improve in muscle contractility.

Tear severity is an additional factor influencing clinical outcomes [16–19]. Massive tears that undergo surgical repair are characterized by having a high re-tear rate [17] due to the difficulty in suturing the torn tendon end onto the original footprint [20–22]. However, our study showed no significant difference in contractility among tear size (*p* = 0.300). Several studies have reported an increase in the applied tension to the tendon during lateral excursion with increasing tear size [20–23]. Chung et al. reported an increase in shoulder abduction power in patients with successful large tear repairs associated with an increase in supraspinatus muscle volume [19]. Our results are consistent with Chung’s outcomes suggesting that a larger excursion of the torn supraspinatus muscle could contribute to a greater increase in contractility.

It also remains controversial if the duration of symptoms is one of the predictors for post-operative function or not [24]. In general, longer duration negatively affected the clinical outcome [25]. However, our regression analysis showed post-operative muscle activity can be estimated by rather the Goutallier stage than the duration of symptoms. Some studies also reported no correlation between post-operative functional outcome and duration of symptoms in the patients with rotator cuff tear [26, 27]. Future studies will be conducted to investigate the relationship between post-operative muscle activity and duration of symptoms, as well as post-operative functional outcomes including muscle strength and range of motion.

Figures 2b, 3b show the individual patient outcomes to emphasize the complicated interaction between fat infiltration, tear size and tendon integrity, and post-operative contractility. While the distribution of *Type III* patients showed four patients classified as stage 1 and three as stage 2 based on the Goutallier stage, *Type III* patients were distributed in every tear size group. This indicates that the post-operative tendon integrity is independent of the initial tear size. In addition, contractility did not improve in patients with *Type III* and *IV* tendons, except for two patients with *Type III* in large and massive tears, as well as stage 1 and 2. Pre-surgical Goutallier stage was an independent factor for post-operative contractility. Thus, *Types III* and *IV* post-operative tendon integrity and pre-operative Goutallier stage 2 and 3 may be critical for predicting post-operative contractility. In other words, although fat infiltration, tear size, and tendon integrity are all confounding factors showing interaction [2, 3], and the influence of tear size still remains controversial, a Goutallier’s stage 2 may be critical to determine if a successful repair can be achieved.

Archiving a Type I Sugaya outcome resulted in a satisfied post-operative outcome in the patients with rotator cuff repair [11]. Increasing tension in torn supraspinatus muscle with increasing fatty degeneration has been commonly known as the negative indicator for post-operative outcome [23]. Considering this with our outcomes, early surgical indication, namely within early Goutallier’s stage, could be effective in obtaining a Type I Sugaya outcome.

In the torn muscle, volume of muscle fibres decreased, fibrosis tissue increased, and the fat tissues fill the space created by reduced muscle volume [28]. This reduced muscle volume, increased fat, and fibrosis tissue may explain the decreased muscle activity correlated with the fat infiltration.

This study contains limitations. First, the distribution of patients based on the Sugaya classification, Cofield classification, and Goutallier stage was not even throughout the various subgroups like previous study [29]. This is due to the intent to properly treat the patients as they presented to the clinic where arthroscopic repairs of the rotator cuff tears were performed as early as possible from the onset. Second, power analysis indicated a small power in subgroup analysis, except for the comparison of pre- and post-operative activity value in the patients with Goutallier stage 1. However, the primary outcome in this study is a multiple regression analysis and the power analysis for this was found to be acceptable (0.63). Third, two patients classified as *Type III*, with large and massive tears, and classified as stage 1 and 2, showed improved contractility post-surgery. One possible explanation for these findings could be an adequate tension present in the repair, which would lead to an improved contractility, even in *Type III* subjects. Future studies should elucidate the relationship between contractility and these degenerative factors. Fourth, it remains unknown whether the contractility measured using RTE is correlated with the strength and functional outcomes. However, we believe that the results in this study showed the usefulness of RTE to monitor the post-operative change in corresponding supraspinatus muscle contractility.

## Conclusion

Multiple regression analysis indicated only the Goutallier stage was a significant independent factor for muscle contractility. Supraspinatus muscle contractility in patients classified as *Types III* and *IV* based on the Sugaya classification tended to decrease post-operatively, regardless of tear size and Goutallier stage; while patients whose contractility increased post-operatively were characterized by having a *Type I* tendon integrity.

## Data Availability

The datasets used and/or analyzed during the current study are available from the corresponding author on reasonable request.
